# Tools of the trade: leveraging 3D *in vitro* models to advance silicosis research

**DOI:** 10.3389/fphar.2026.1782408

**Published:** 2026-05-04

**Authors:** Ellen Donohoe, Sakthi Priya Selvamani, Vivek Dharwal, Richard Zelei, Anthony Linton, Deborah Yates, Elham Hosseini-Beheshti

**Affiliations:** 1 Asbestos and Dust Diseases Research Institute, Concord, NSW, Australia; 2 Faculty of Medicine and Health, University of Sydney, Sydney, NSW, Australia; 3 Concord Repatriation General Hospital, Sydney, NSW, Australia; 4 Holdsworth House Medical Practice, Darlinghurst, NSW, Australia; 5 School of Clinical Medicine, University of New South Wales, Sydney, NSW, Australia; 6 Respiratory and Sleep Medicine, Macquarie University Hospital, Macquarie University, Sydney, NSW, Australia

**Keywords:** 3D models, drug development, occupational disease, pulmonary fibrosis, respirable silica, silicosis

## Abstract

Silicosis is an occupational fibrotic lung disease representing a major public health crisis, and places a significant burden on patients and healthcare systems. There are no curative treatments for patients with silicosis, and available anti-fibrotic agents have not been shown to reduce disease progression. Enhanced understanding of the cellular mechanisms governing silicosis is required to establish biomarkers of disease onset and progression to allow for early detection, timely intervention, and development of improved therapeutics. Physiologically relevant 3D *in vitro* models enable investigation into mechanisms driving disease onset and progression, offering robust platforms for biomarker identification and drug screening, as well as toxicological assessments of silica-containing materials. Importantly, different models can be leveraged to recapitulate the various features of disease, depending on the specific research objective. This review highlights the current landscape of 3D models applied to silicosis research, highlighting innovative tools that can be harnessed to deepen our understanding of disease pathobiology, improve the drug development pipeline, and ultimately improve patient outcomes. We explore the limitations and opportunities of different modelling platforms specific to silicosis, and highlight translational opportunities for using human-centered modelling systems to evaluate the hazards of silica-containing materials and in representing varied occupations and exposure cohorts.

## 1 Introduction

Silicosis is an irreversible occupational lung disease caused by inhalation of respirable silica particles (Leung et al., 2012). Once inhaled, silica particles reach the small airways and cannot be effectively removed, triggering persistent inflammation and fibrotic remodelling, thereby reducing lung function and significant disability (Liu et al., 2023; Zhang et al., 2025b). A recent study reported that 230 million people worldwide are exposed to respirable silica, with an estimated 12,900 deaths due to silicosis, and prevalence of silicosis is rising on a global scale (Liu et al., 2023; Zhang et al., 2025b). In Australia, exposure in the artificial stone industry triggered a resurgence of silicosis, leading to the world’s first national ban on artificial stone (Barnes and Chambers, 2024). However, substantial silica exposure persists in industries such as mining, tunnelling, quarrying, and construction (Hoy and Chambers, 2020; Leung et al., 2012; Yang et al., 2025). The true burden of silicosis and other silica-related diseases is likely to be grossly underestimated, due to insensitive methods of detection, lack of awareness, and the variable latency from exposure to symptomatic presentation (Liu et al., 2023).

Despite regulatory requirements for health monitoring, many cases of silica-induced lung disease remain undiagnosed, particularly in the early stages of disease when workers are often asymptomatic. This may result in detection of disease at advanced stages, which poses significant challenges for effective treatment (Hoy and Chambers, 2020; Barnes and Chambers, 2024; Li et al., 2022). Current treatment options are limited, aiming to reduce symptoms and slow progression of disease, but do not offer restoration of lung function or reversal of fibrosis in established disease (Li et al., 2023; Handra et al., 2023). Silica-induced lung diseases place a significant burden on individuals, families, and the healthcare system (Zhang et al., 2025b). In 2012–13 alone, occupational lung diseases, including silicosis, cost the Australian economy $61.8 billion in direct healthcare, specialist care, and management, alongside indirect costs from lost productivity, income replacement, and workers’ compensation (Safe Work Australia, 2015).

In order to mitigate this urgent healthcare crisis and improve patient outcomes, further insights into the underlying pathophysiology and factors governing disease onset and progression are needed to allow for earlier detection and timely intervention. Identification of potential therapeutic targets based on cellular drivers of disease should also pave the way for improved therapeutics. Given that tissue biopsies are infrequently performed for diagnosis of silicosis, there is a critical need to develop robust and disease-specific pre-clinical models that accurately replicate the silicotic microenvironment of the lung.

Several experimental animal models have been developed for silicosis research (Lam et al., 2025; Peng et al., 2022; Martinez-Lopez et al., 2023). However, physiological differences limit the utility of animal models for studying silicosis, often resulting in poor clinical translatability, and failure to exhibit hallmarks of human disease (Walters and Shukla, 2021). In addition, the poor success rate of drug candidates targeting pulmonary fibrosis in clinical trials is largely attributed to limitations in pre-clinical efficacy screening and lack of relevance to human physiology (Kola and Landis, 2004; Dowden and Munro, 2019; Sun et al., 2022; Fogel, 2018; Loskill et al., 2021). Recent advances in three-dimensional (3D) modelling have significantly enhanced our ability to recapitulate complex respiratory disease processes, offering physiologically relevant platforms for investigating disease mechanisms and predicting therapeutic responses (Wallace et al., 2025; Bittner et al., 2018; Hayward et al., 2021; Strikoudis et al., 2019; Sachs et al., 2019). Although not widely utilised in silicosis research to date, 3D models hold significant potential to aid in elucidating the cellular and molecular drivers of silica-induced inflammation and fibrosis. Emerging technologies such as human-on-a-chip systems and bioprinting further enable the recreation of lung-specific architecture and dynamic microenvironments, improving fidelity and scalability (Huh et al., 2010; Liu et al., 2025; Kang et al., 2021). This review explores the current landscape of advanced *in vitro* models applied to silicosis research, highlighting innovative 3D tools that can be harnessed to deepen our understanding of disease pathobiology, and improve translational relevance.

## 2 Silicosis pathogenesis and preclinical modelling: lessons from 2D cultures and animal models

Silicosis, as mentioned earlier, is a chronic fibrotic disease caused by exposure to respirable silica particles. Based on the duration and intensity of exposure, the disease is characterised as acute, accelerated, or chronic. While we do not yet fully understand the pathogenesis of disease, particularly the exact molecular mechanisms driving fibrosis and the temporal sequence of immune responses, the disease involves overlapping phases of parenchymal changes, inflammation, and tissue remodelling (Yang et al., 2025). Briefly, the innate respiratory defence consists of nasal hairs, the mucociliary system, and resident alveolar macrophages (AMs), which act as the first line of defence. A large body of evidence shows that chronic exposure to silica particles can compromise these protective mechanisms. Studies utilising bronchoscopy samples and rodent models have reported that exposure to respirable silica reduces the number of cilia in the airway epithelium, and drives fibrotic remodelling, evidenced by altered ECM homeostasis, increased collagen deposition and elastic fibres, resulting in tissue stiffening (Regland et al., 1976; Li et al., 2020; Yu et al., 2020; Faffe et al., 2001; Delgado et al., 2018; Sun et al., 2025). Silica exposure is also associated with ultrastructural abnormalities of the airway, including absence of central pair microtubules, disorganised microtubules and clusters of axonemes in cilia, as well as mucus hypersecretion (Regland et al., 1976; Li et al., 2020; Yu et al., 2020). AMs maintain lung immune homeostasis, and silica particles entering the alveolar spaces are internalise by AMs *via* scavenger receptors in an attempt to degrade the particles in phagolysosomes (Handra et al., 2023). Chronic silica exposure overwhelms this defence, leading to AM death by apoptosis and necrosis, and release of factors involved in recruitment of monocytes, neutrophils, mast cells, and adaptive immune cells, triggering multiple signalling pathways (Liu et al., 2024b). This initiates epithelial-mesenchymal transition (EMT), a process in which epithelial cells acquire mesenchymal features. Among others, TGF-β signalling is a key driver of this (Handra et al., 2023; Qin et al., 2021; Merchant et al., 1990). This ultimately results in irreversible damage to lung tissues and deterioration of lung function (Yang et al., 2025).

Various studies have investigated the influence of silica exposure in 2D cell culture models, providing insights into their biological effect on specific cell types, and the relationship between toxicity and exposure dose. A wide range of cell types have been utilised to investigate the toxicity of respirable silica, including mouse- and rat-derived macrophages, T lymphocytes, alveolar epithelial cells, fibroblasts, and human-derived bronchial and alveolar epithelial cells, macrophages, and fibroblasts (Thibodeau et al., 2004; Perkins et al., 2012; Merchant et al., 1990; Lv et al., 2022; Joshi and Knecht, 2013; Cho et al., 1999; Cao et al., 2017; Qu et al., 2023; Jin et al., 2021; Liu et al., 2022b). These studies informed silica-induced disease mechanisms such as apoptosis, autophagy, fibrosis, senescence, EMT, endothelial-mesenchymal transition (EndMT), lung damage, and carcinogenesis (Venkatesan et al., 2022; Tsugita et al., 2017; Thibodeau et al., 2004; Qin et al., 2021; Perkins et al., 2012; Merchant et al., 1990; Lv et al., 2022; Liu et al., 2017; Liang et al., 2016; Lee et al., 2009; Joshi and Knecht, 2013; Cho et al., 1999; Cao et al., 2017; Qu et al., 2023; Herseth et al., 2008; Yang et al., 2016). However, there are numerous variables between studies that drive inconsistent reporting and poor reproducibility which hinders progression in identifying key attributes responsible for silica-induced disease. Silica treatment *in vitro* has been reported using both surface area-based (µg/cm^2^) and volume-based (µg/mL) dosing methods, with concentrations varying substantially and ranging from 2.5 μg/cm^2^ to 200 μg/cm^2^, and 25 μg/mL to as high as 1,000 μg/mL, with exposure duration ranging from 0.5 h to 72 h based on the cell type and biological condition investigated (Venkatesan et al., 2022; Tsugita et al., 2017; Thibodeau et al., 2004; Qin et al., 2021; Perkins et al., 2012; Merchant et al., 1990; Lv et al., 2022; Liu et al., 2017; Liang et al., 2016; Lee et al., 2009; Joshi and Knecht, 2013; Cho et al., 1999; Cao et al., 2017; Hetland et al., 2001). While most reports have highlighted a wide range of silica doses administered in vitro studies, there is a lack of information on the quantification of deposited or internalized silica at the cellular level. The discrepancy among the administered, internalized dose, and dosimetry properties of silica in submerged cell culture systems could be influenced by differences in cell culture media, particle size, and their distribution (Giorgi et al., 2021). When resuspended in a solution, silica particles may possess different physicochemical characteristics compared to freshly fractured dust. The dampening effect of serum on silica particle toxicity, whereby serum creates a corona complex around the silica particles and reduce their toxicity, has been also reported, but rarely reported on in pre-clinical toxicology studies (Böhmert et al., 2020; Docter et al., 2014). While research has also shown that ‘old’ silica dust is less toxic compared to freshly milled particles, the latency and storage conditions between fractionation and experimental exposure in a pre-clinical model is also under-reported (Vallyathan et al., 1988; Castranova et al., 1996). These variabilities impact particle kinetics and toxicodynamics in *vitro* models, making inter-study comparisons and reproducibility challenging. In order to better identify critical physicochemical attributes driving silica-induced disease and produce pre-clinical studies reflective of real-world exposure scenarios, transparency and improved reporting methods are needed.

Concerns surrounding the clinical applicability of 2D cultures have emerged in recent years, with significant limitations in terms of biological relevance to human disease. Cells grown on flat plastic surfaces exhibit altered cellular phenotypes due to the lack of microenvironmental matrix and spatial organisation found *in vivo*. A growing body of evidence demonstrates that 2D models often fail to accurately capture responses observed *in vivo* (Engler et al., 2006; Molnar and Labouesse, 2021; Kapałczyńska et al., 2018). 2D cultures are also limited by shorter cultivation time which limits the extent of cellular changes observed, emphasising the necessity for long-term models to simulate changes encompassing the early inflammatory to fibrotic stages of silicosis (Jensen and Teng, 2020; Molnar and Labouesse, 2021). In addition, an important limitation of 2D culture models is their inability to capture chronic inflammatory and fibrotic processes which are hallmarks of silicosis progression. In particular, the involvement of the immune system, such as repeated phagocytosis of silica particles by AMs and immune activation culminating in progressive fibrotic remodelling, are difficult to capture in 2D cell culture systems (You et al., 2024; Rocha et al., 2012; Jensen and Teng, 2020).

Animal models of silicosis have provided valuable insights into disease pathobiology, discussed in depth in recent reviews (Martinez-Lopez et al., 2023; Amudha and Udayakumari, 2024). These models have played an important role in advancing our understanding on how silica concentration and duration of exposure correlate with disease severity, and have also been leveraged to better understand the influence of genetics in disease phenotype, and evaluation of potential therapeutics (Peng et al., 2022; Martinez-Lopez et al., 2023; He et al., 2024; Wu et al., 2025; Liu et al., 2024a; Rocha et al., 2012). However, significant anatomical and physiological differences between animals and humans limit the relevance and utility of these models for studying human diseases, often resulting in poor clinical translatability and less predictive validity (Crystal et al., 2008; Martinez-Lopez et al., 2023; Walters and Shukla, 2021; Padmanabhan et al., 2019). Furthermore, animal models often fail to replicate key hallmarks of human disease, raising concerns about their suitability for investigating the molecular mechanisms underlying silicosis. Therefore, improved pre-clinical models that closely resemble the complex structure, function, and microenvironment of human lungs are required to better understand silica-induced pathobiology and clinical manifestations, thereby to facilitate drug discovery and validation (Sacchi et al., 2020; Nizamoglu et al., 2023).

## 3 The evolution of 3D *in vitro* models and their relevancy in silicosis research

3D culture techniques have accelerated disease modelling and drug screening (Nizamoglu et al., 2023; Jensen and Teng, 2020). These advanced *in vitro* models allow for in-depth understanding of disease pathogenesis by enabling uniquely controlled conditions, allowing investigation into subtle changes in microenvironment such as matrix composition and stiffness (Bittner et al., 2018; Grigoryan et al., 2019; Sacchi et al., 2020). 3D models can replicate tissue architecture, oxygen and nutrients gradients, and the dynamics of cell-cell and cell-matrix interactions, all of which are critical for understanding disease development and progression (Sacchi et al., 2020). Furthermore, 3D models can be scaled for high-throughput, reproducible, and physiologically relevant drug screening platforms that accelerate both the drug-development pipeline and clinical candidate selection (Sharma et al., 2024; Matera et al., 2021). As regulatory bodies shift towards non-animal innovation for toxicity assessments and drug screening, it will be critical to improve our *in vitro* models for silica-induced disease (Han, 2023). While 3D models have been extensively investigated in various respiratory diseases, their application in silicosis remains under researched (Li et al., 2024a; Song et al., 2018; Song et al., 2021; Stroulios et al., 2022; Jose et al., 2020). The following sections will discuss the current studies that have employed air-liquid interface systems (summarised in Table 1) and 3D cell culture systems (summarised in Table 2) for silicosis research. We also highlight advanced technologies for next-generation model development, and identify opportunities and challenges associated with these modelling platforms in the context of silicosis.

**TABLE 1:** Summary of studies to-date using ALI models to assess silica-induced inflammation and fibrosis.

Species	Cell source	Silica type, characteristics, and source	Silica concentration, exposure frequency, and duration	Main findings	Ref.
Mouse	Peripheral lung tissue	Crystalline silica (Sigma-Aldrich^*^) Purity: 99% Particle diameter: 0.5–10 μm	50 μg/cm^2^ silica suspension for 24 h pre-ALI Endpoint analysis day 28	Silica-induced NLRP3 inflammasome activation disrupted the epithelial architecture, abnormal mucociliary differentiation and mucus hypersecretion, induced ciliary hyperplasia, and EMT.	Zhou et al. (2023)
Human	A549	Aerosil200 amd SiO2-50 nm amorphous silica (postnova analytics; Z-PS-SIL-GFP-0.07) Particle diameter: 50 nm	Deposited using Vitrocell® aerosoliser Aerosil200: 52 ± 26 μg/cm^2^ deposited after 5 h SiO2-50 nm: 117 ± 46 μg/cm^2^ after 7 h Submerged conditions: 15.6 μg/cm^2^ Endpoint analysis 24 h	Cells exposed at the ALI were less sensitive to silica nanoparticles, evidenced by reduced cytotoxicity and IL-8 levels	Panas et al. (2014)
Human	MatTek EpiAlveolar™	DQ12 quartz^*^ 87% crystalline silica and amorphous silica with kaolinite impurities Particle diameter: ≤5 μm	∼0.2 μg/cm^2^ DQ12 deposited using Vitrocell® Cloud aerosoliser 5 days per week for 3 consecutive weeks Total deposition after 3 weeks ∼3 μg/cm^2^ Endpoint analysis day 21	Exposure to DQ12 did not induce cytotoxicity, and did not affect epithelial cell tight junction formation. Secretion of TNF-α, IL-1β, TGF-β, IL-18 and IL-6 was dependent on the presence of macrophage. Significant increase in fibronectin production at day 21, and in COL1 production at days 1, 14, and 21, but not observed when macrophage were added	Barosova et al. (2020)
Human	A549 THP-1	DQ12 quartz^*^ Particle diameter: 93% < 1.0 μm	10, 20, or 30 μg/cm^2^ DQ12 deposited using Vitrocell® Cloud aerosoliser, exposed for 24 h Endpoint analysis 24 h	DQ12 did not induce cytotoxicity or markers of oxidative stress dose-dependent increase in GMCSF, GCSF, IL-1β and IL-6. No change in IL-8, TNFα, or IL-18 expression	Bredeck et al. (2024), Bredeck et al. (2023)
Human	A549 THP-1	Min-U-Sil5 quartz particles (U.S. Silica) Purity: 99.4% Particle diameter: 0.5–2 µm	Submerged exposure 15, 30, or 60 μg/cm^2^ ALI culture exposed to ∼16, 30, 60 μg/cm^2^ using Vitrocell® Cloud aerosliser Endpoint analysis 24 h	Min-U-Sil5 did not induce a cytotoxic response in A549 mono or A549/THP-1 co-cultures at ALI. Dose-dependent increase in IL-8 in co-culture ALI, but not in A549 monoculture. Authors suggest submerged silica exposure is more sensitive than aerosolised delivery to ALI.	Friesen et al. (2022)
Human	NHBE A549 BEAS-2B THP-1 Calu-3	DQ12 (institute of occupational Medicine, Edinburgh, United Kingdom) - crystalline, 0.4–1.6 µm diameter NM-203 (European Commission) - pyrogenic amorphous, 74 nm diameter Silica-Std (nouryon) - colloidal amorphous, 17 nm diameter Silica-Silane (nouryon) - silane surface functionalized colloidal, 17 nm diameter	Deposited up to 4.4 μg/cm^2^ using Vitrocell® Cloud aerosoliser at ALI, or 0–125 μg/mL in submerged conditions Endpoint analysis 24 h	Pre-regulatory hazard ranking of particles was based on cell viability, oxidative stress, haemolysis, and secretion of IL-8, MCP-1, IL-6, TNFa. DQ12 did not exhibit toxicity, differing from well-established literature, suggesting a chronic and persistent exposure model may be needed to more accurately predict hazard rankings	Ruijter et al. (2025)

* Source/catalogue number not specified. ALI, Air-Liquid Interface; EMT, Epithelial to mesenchymal transition; NHBE, Normal human bronchial epithelial cells; NP, nanoparticle.

**TABLE 2:** Summary of studies to-date using 3D *in vitro* models to assess silica-induced inflammation and fibrosis.

Culture system	Species	Cell source	Silica type, characteristics, and source	Silica concentration, exposure frequency, and duration	Main findings	Ref.
PCLS	Human	Transbronchial biopsy sample (lung cancer diagnosis)	Commercial raw silica microparticles (dost kimya, Turkey^*^) Average particle diameter: 960 nm	800 μg/mL under dynamic conditions on shaker Endpoint analysis 24 h	Deteriorated epithelial barrier integrity and morphological structure. Reduced adhesion marker expression (ACE2, β-catenin, E-cadherin) and increased pro-inflammatory marker expression (IL-1α, IL-6, IFN-_Ɣ_, iNOS and CD80)	Goksel et al. (2024)
PCLS	Mouse	WT and Pr2x7 KO lung tissue AMJ2-C11	Nano-silica (Sigma-Aldrich^*^) Spherical, porous Average particle diameter: 5–15 nm	1,600 μg/mL in a roller system for 3 mL volume without macrophage, 5 mL volume with macrophage co-culture Endpoint analysis 72 h	AMs influence the early inflammation of the lung Silica-induced inflammation is reduced in P2rx7^−/−^ mice compared to wild type mice	Hofmann et al. (2015)
Matrigel® matrix (multicellular)	Human Mouse	RAW264.7: A549: MRC-5 (5:3:2 ratio)	Silica (Jinhao co. Ltd.^*^) Purity: 99% Particle diameter: 20–50 nm	25, 50, 100 μg/mL single exposure Endpoint analysis 24 h	Silica exposure caused NFkB-dependent inflammatory response, EMT and myofibroblast transdifferentiation. Increase in TNF-α and IL-1β was greater in 3D than in 2D model	Yang et al. (2023)
Decellularized lung matrix-derived spheroids (monoculture)	Mouse	RAW264.8 NIH/3T3	Not specified*	Macrophage exposed to 50 μg/mL silica for 24 h Fibroblast spheroids cultured in macrophage secretome for up to 14 days	Silica-induced collagen deposition mimics development of silicotic nodules and leads to an apoptosis-resistant myofibroblasts acting through the Nrf2/Bax pathway	Xue et al. (2024)
Collagen matrix (monoculture)	Human	WI38-VA13	Min-U-Sil 5 (U.S. Silica) Purity: 99.2% Average particle diameter: 1.6 µm	1 μg/mL single exposure Endpoint analysis 48 h	Silica exposure induced myofibroblast differentiation and collagen deposition, as well as fibrotic contraction	Hindman and Ma (2018)
Collagen matrix (monoculture)	Human	HPF-a	Silica (sigma-aldrich; S5631) Purity: 99% Particle diameter: 1–5 um	25, 50, 100, 200 μg/cm^2^ Endpoint analysis 24 h	Induction of MCPIP1 in a MAPK- and PI3K/Akt-dependent manner, with peak MCPIP1 expression at 3–6h, and sustained increase in α-SMA. Increased fibroblast migration *via* the MCP-1/CCR2 pathway	Liu et al. (2016) Liu et al. (2015)
Organoid	Mouse	Peripheral lung tissue	Crystalline silica (sigma^*^) Purity 99% Particle diameter 0.5–10 μm	50 μg/cm2 silica, single exposure Endpoint not specified	Silica-induced NLRP3 inflammasome activation prevented lung organoid development and suppressed proliferation and pluripotency	Zhou et al. (2023)
AEB-on-a-chip	Human	Calu-3	Commercial raw silica microparticles (dost kimya, Turkey^*^) Average particle diameter: 960 nm	800 μg/mL under dynamic or static conditions Endpoint analysis 24 h	Increased barrier permeability, decreasing cell adhesion-barrier markers, impaired cell viability and increased expression of pro-inflammatory markers Changes more dramatic in dynamic conditions compared to static	Goksel et al. (2024)
Lung-on-a-chip	Human	NCI-H441 A549 Microvascular endothelial cells	Ludox LS colloidal silica (sigma-aldrich) Particle diameter: 12 nm	100 μg/mL silica delivered at the ALI Endpoint analysis 5 h	Cyclic mechanical strain accentuates silica-induced toxicity and inflammatory response. Mechanical strain enhances epithelial and endothelial uptake of silica nanoparticles and stimulates transport into underlying microvascular channel	Huh et al. (2010)

* Source/Catalogue number not specified. AEB, Alveolar epithelial barrier; AM, Alveolar macrophage; EMT, Epithelial to mesenchymal transition; PCLS, Precision cut lung slice.

### 3.1 Air-liquid interface (ALI)

In ALI culture, the basal surface of cells is in contact with growth medium, representing the circulatory system, and the apical surface is exposed to air. Many studies have employed ALI cultures using primary epithelial cells or immortalised epithelial cell lines to mimic the phenotypic features of human airways, including a columnar layer, tight junction formation, multilayered cell development, and mucin expression (Kreft et al., 2015; Grainger et al., 2006; Forbes et al., 2003; Baldassi et al., 2021). ALI has been extensively used to study the effects of particulate matter (PM) particles, which share several key features with silica, including particle size, deposition patterns, and the capacity to induce inflammation, disrupt airway epithelial integrity and cause oxidative stress (Vanka et al., 2022). Parameters such as mitochondrial dysfunction, oxidative stress, inflammation, and autophagy following PM exposure have been studied using these models (Vanka et al., 2022). Given the similar exposure kinetics and physicochemical characteristics, these studies can inform on the utility of ALI cultures for silicosis research and investigating the biological effects of inhaled silica particles on the epithelial barrier and mucociliary system. Zhou et al. have used peripheral lung tissue from mouse lungs to generate a pseudostratified columnar ciliated epithelium that included MUC5AC^+^ goblet cells to investigate various pathological features of silica-induced injury, including NLRP3 inflammasome activation, pyroptosis, mucus production, collagen-driven remodelling, abnormalities in mucociliary differentiation, and multiciliogenesis using ALI (Zhou et al., 2023).

A major advantage of the air-exposed interface of ALI is the ability to investigate the biological effects of silica using nebulisation or aerosolisation systems. This approach enables the deposition of materials in a manner more representative of real-world exposure, reflecting interactions with the lung that occurs when such particles are inhaled. Studies have shown that in submerged 2D cultures the sedimentation-diffusion equilibrium of nanoparticles depends on factors such as temperature and colloidal stability. This can result in a significant difference between the target dose of these particles and the administered dose (Giorgi et al., 2021). Deposition at the ALI also mitigates potential corona complex formations that alter particle toxicodynamics, as discussed in the previous section (Docter et al., 2014; Böhmert et al., 2020). Friesen et al. conducted a comparative study to evaluate the toxicity of quartz after 24 h exposure using A549 epithelial cells, differentiated THP-1 macrophages, and A549/THP-1 co-cultures in submerged and ALI culture settings (Friesen et al., 2022). In this study, quartz was found to induce dose-dependent cytotoxic and pro-inflammatory effects in submerged cultures, with THP-1 cells showing greater susceptibility than A549 cells. Notably, IL-8 release was enhanced in co-cultures exposed to quartz, highlighting the importance of cell-to-cell interactions in pro-inflammatory signalling. However, ALI cultures exposed to aerosolised quartz resulted in minimal cytotoxicity and lower IL-8 secretion, suggesting a reduced response in conditions that more closely mimic physiological exposure. The authors of this study suggested that submerged models are more sensitive to quartz-induced toxicity, while ALI systems can better replicate *in vivo* exposure, underscoring the critical role of model selection in particle toxicology research. Similar findings were also reported in other studies using A549, Calu-3 and THP-1 cells, showing a more pronounced response to quartz/silica particles after 24 h of exposure in submerged conditions compared to ALI cultures (Ruijter et al., 2025; Panas et al., 2014).

ALI can also be maintained for several weeks, allowing investigation into repeated exposure of silica. Barosova et al. developed an alveolar model (EpiAlveolar™) to predict long-term responses to aerosols, consisting of human endothelial cells seeded on the basal side of a microporous membrane, epithelial cells and fibroblasts seeded on the apical side, and grown at the ALI (Barosova et al., 2020). Their study simulated occupational exposure by subjecting the models to silica particle deposition at physiologically realistic concentrations every working day for three consecutive weeks using an automated aerosoliser. The authors observed dynamic changes in cytokine secretion profiles over the course of the study and noted increased fibronectin production at day 21, emphasising the need for repeated exposure models for robust prediction of inflammatory and fibrotic response to respiratory hazards such as respirable silica. When exposed to TGF-β, the EpiAlveolar™ model generated a nodular structure with tissue contraction (Barosova et al., 2020). While it was not reported that this occurred with exposure to quartz, ALI may represent a method to replicate silica-induced fibrotic nodes seen in patients.

One limitation of the ALI culture is its inability to mimic complex epithelial and immune cell interactions. As discussed, immune and epithelial interactions play a critical role in initiating EMT pathways that result in irreversible tissue remodelling. While studies have utilised multicellular ALI co-cultures, these models are incorrectly oriented, as immune cells remain in the basolateral compartment and exhibit limited migration (Castellani et al., 2018; d'Angelo et al., 2018). Additionally, a lack of vascularisation and stromal support limits the application of these systems. Achieving a fully differentiated pseudostratified columnar ciliated epithelium requires prolonged culturing, making these models laborious and difficult to scale. In addition, when using ALI to assess the fibrotic effects of silica particles, researchers have noted the potential for tissue contraction that may cause detachment from the insert membrane (Barosova et al., 2020).

Overall, ALI offer advantages for evaluating toxicity in relation to epithelial integrity, mucus production, and structural abnormalities. Due to the lack of complete physiological complexity for silica-induced lung disease, their use for high-throughput drug screening and evaluation of emerging therapeutics for silicosis patients may be limited.

### 3.2 Hydrogel scaffolds

The lung ECM is comprised of heterogenous aggregates of proteins such as collagens, elastin, glycoproteins and proteoglycans, that offer structural support and mechanical stability to cells. ECM also influences cell survival, growth, and phenotype, and constantly undergoes remodeling to control tissue homeostasis and wound repair (Bonnans et al., 2014; Hansen et al., 2015; Li et al., 2024a). During pathogenic fibrogenesis, however, the lung’s ECM undergoes significant changes including increased stiffness and altered composition, which contributes to disease progression by promoting fibroblast activation, myofibroblast apoptosis resistance, and tissue remodeling through excessive ECM deposition, creating a pathogenic feedback loop and destruction of the airway architecture (Bonnans et al., 2014; Upagupta et al., 2018). Diseases such as IPF, COPD and asthma are marked by abnormal ECM deposition resulting in fibrosis, stiffening of the lungs, and impaired gas exchange (Bonnans et al., 2014; Hansen et al., 2015; Upagupta et al., 2018). Extensive tissue remodeling and ECM depositions are also associated with silicosis, necessitating the development of models that can replicate these processes (Sun et al., 2025; Hansen et al., 2015). Advances in understanding the role of the ECM in fibrosis have also opened pathways for ECM-targeted therapeutics which may provide clinical benefit for patients with silicosis (Huang et al., 2021; Mayorca-Guiliani et al., 2025). However, initial efficacy assessment of such drugs for such patients will require a robust pre-clinical model that incorporates the ECM.

Individual ECM components or derivatives such as collagen, matrigel, elastin, and hyaluronic acid are widely studied to form hydrogels that mimic the native ECM. Studies using collagen matrices have established silica-induced matrix remodeling and excessive matrix deposition by myofibroblasts, and have identified an association between MCPIP1 signaling and fibroblast migration following silica exposure (Hindman and Ma, 2018; Liu et al., 2016; Liu et al., 2015). Liu et al. found that fibroblast response to silica differs when they are cultured in 2D compared to those embedded in a 3D collagen matrix, highlighting the importance of 3D models to mimic cell-matrix adhesion and interaction observed *in vivo* environments (Liu et al., 2016). Studies have also shown that silica toxicity is dependent on the type of scaffold used to culture the exposed cells (Kim et al., 2022). Kim et al. demonstrated that silica nanoparticles were non-toxic to HepG2 cells when cultured in matrigel but were toxic when alginate or collagen I scaffolds were used (Kim et al., 2022). This highlights that silica-induced toxicity and fibrosis may vary depending on the culture system and should be carefully considered when using scaffolds for silicosis research.

Current hydrogel models of silica-induced disease are limited by cellular complexity and short-term cultivation, as well as inherent mechanical weaknesses, and simple hydrogel structures do not capture the physiological complexity of the ECM and its macrostructure. Sourcing native lung ECM through allogeneic or xenogeneic lung decellularization (dECM), whereby the cellular content of the lung is removed leaving behind intact ECM as a substrate for 3D cell culture, has been investigated in several pulmonary fibrosis studies (Li et al., 2024a; Wang et al., 2023; de Hilster et al., 2020). This technique offers a more robust representation of organ-specific biochemical and biophysical complexity, which is more ideal for complex diseases such as silicosis. Xue et al. developed a novel 3D hydrogel model using decellularized mouse lung matrices as scaffolds coupled with self-assembled fibroblast spheroids, to simulate the development of silicosis and evaluate the role of fibroblasts in disease progression (Xue et al., 2024). Secretome from silica-stimulated macrophages was found to induce fibroblast-mediated collagen secretion and deposition through the Nrf2/Bax pathway. Interestingly, the authors observed a gradual aggregation of the deposited collagen into concentric patterns within the spheroids similar to the pathological structure of the silica nodules observed *in vivo*. Such models may be valuable for studying therapeutic drug penetrance into nodules in silicosis patients, and provide insights into pathways regulating nodule development, allowing identification of novel targets for drug development. Decellularization of human lung tissue is well described, although not yet investigated in the silicosis field of research (de Hilster et al., 2020).

Opacity challenges and autofluorescence presents challenges for imaging dECM hydrogels in order to capture how cells interact with their microenvironment (Merna, 2025; Tsuchiya et al., 2014). It should also be noted that decellularization using harsh chemicals can damage ECM structure and result in loss of proteoglycans, which may limit the physiological robustness of this technique for preserving native lung ECM (de Hilster et al., 2020; Tsuchiya et al., 2014). Caution should also be encouraged when using rodent lung ECM, as there are significant differences between rodent and human lung tissue (Stucki et al., 2024). Rodent lung tissue has lower absolute stiffness and higher viscoelasticity, and reduced ECM complexity and compactness to accommodate their high breathing rates (Guo et al., 2022). Inferring results from studies using ECM that is not physiologically relevant may result in poor clinical translatability, and should be carefully considered when developing such models for silicosis research. Hydrogel-based approaches are also prone to batch-to-batch variability resulting in poor reproducibility (Hansen et al., 2015; Li et al., 2024a). Hydrogel scaffolds also do not provide an ALI arrangement or dynamic flow (Sedláková et al., 2019; El-Sherbiny and Yacoub, 2013). Therefore, the utility of these models may be restricted to studies where ECM remodeling and mechano-transduction in silica-induced disease is central to the research question.

### 3.3 Precision cut lung slices (PCLS)

PCLS are thin, uniform portions of lung tissue derived from humans or animals that retain the native lung cellular diversity and 3D architecture. PCLS from healthy lung tissue have allowed toxicological assessments of environmental pollutants such as bushfire smoke, pesticides, and cigarette-smoke extract in anatomically relevant settings (Shrestha et al., 2022). These studies have identified key cell types and molecular signatures that drive fibrosis in diseases such as COPD and IPF, highlighting the potential of PCLS to study the development and progression of silicosis (Liu et al., 2022a; Hansen et al., 2016; Koziol-White et al., 2024). Silica microparticles have been shown to diffuse into PCLS from healthy human bronchial biopsies, inducing cytotoxicity and inflammatory signaling evidenced by elevated IL-6, IL-1α, IFNγ and CD68 expression (Goksel et al., 2024). Impaired morphological structure and deteriorated barrier integrity are also observed in silica-exposed PCLS (Goksel et al., 2024). Another study by Hoffman et al. utilized healthy murine PCLS co-cultured with immortalized AMs to evaluate the role of P2rx7 in the early inflammatory response to amorphous nano-sized silica (Hofmann et al., 2015). The authors found reduced cell damage, apoptosis and inflammatory cytokine release in P2rx7^−/−^ PCLS compared to wild type lung tissue. Use of PCLS significantly reduces the number of animals required compared to *in vivo* studies, and PCLS from genetically modified animals may provide useful information on specific pathways involved in silica-induced inflammation and fibrosis. However, while animal-derived PCLS may circumvent the limited availability of healthy human lung tissue for slicing, the well documented differences between animal models and human anatomy and physiology limits clinical translatability of studies using xenogeneic PCLS (Stucki et al., 2024; Guo et al., 2022; Kola and Landis, 2004). Alternatives to healthy human tissue, such as tumor-free tissue from lung cancer resection surgeries of patients without a fibrotic lung disease diagnosis, should be investigated as substitute sources (Preuss et al., 2022; Liu et al., 2022a).

PCLS can also be prepared from patient biopsy samples and utilized as an *ex vivo* model to simultaneously evaluate the efficacy of multiple therapeutic interventions in a single patient’s tissue (Mercer et al., 2016; Lang et al., 2023; Hansen et al., 2016; Huang et al., 2021). Studies have shown cell-cell and cell-matrix interactions are largely conserved in patient-derived PCLS, allowing for predictive response to therapeutics and personalized medicine (Lang et al., 2023; Mercer et al., 2016; Huang et al., 2020; Hansen et al., 2016). To date, there are no studies examining PCLS derived from the lungs of silicosis patients. While patient biopsy is not routine for diagnosis and obtained explants may be limited to end-stage disease, new techniques such as endoscopic bronchial ultrasound and cryobiopsy may enable isolation of silicotic tissues at earlier stages of disease (Alsafadi et al., 2017). Therefore, PCLS represents a platform for personalized medicine in silicosis and testing emerging or repurposed therapeutics.

Aside from tissue procurement issues, there are technical limitations that should also be considered when selecting PCLS as a model for silicosis (Preuss et al., 2022; Liu et al., 2022a). It is important to acknowledge that *ex vivo* tissues may rapidly change once placed in culture and are, to some extent, representative only of the cell populations existing in the lung tissue at the time of extraction (Preuss et al., 2022; Liu et al., 2022a). Long-term PCLS cultivation is a significant challenge, with continuous loss of cellular viability, alveolar integrity and reduced ciliary beat in the small airways after the first week (Temann et al., 2017; Neuhaus et al., 2018; Preuss et al., 2022). Transcriptomic analysis has also revealed increased immune response and reduced metabolic activity within the first 24 h of PCLS generation (Temann et al., 2017; Neuhaus et al., 2018; Preuss et al., 2022). It should be noted that studies utilizing healthy PCLS to study silica-induced injury have been short-term (up to 72 h), and while useful in understanding the early inflammatory response, it does not add to the knowledge on fibrosis or silicotic nodule development. Issues with long-term cultivation may limit the usefulness of healthy PCLS in modelling silica-induced fibrosis where chronic exposure to silica may be necessary for true recapitulation of disease and longer-term disease development beyond the early inflammatory stage (Preuss et al., 2022; Liu et al., 2022a). While there is great potential for PCLS platforms in advancing research into silicosis, further efforts to optimize culture conditions will be essential.

### 3.4 Organoids

Lung organoids are self-organizing multicellular 3D cultures, with a spatial arrangement that mimics the *in vivo* structure and function of the airways. Derived from stem/progenitor cells including those from adult, embryonic, and induced pluripotent stem cells (iPSCs), organoids model multi-lineage lung biology (Zhang et al., 2025a). Pluripotent cells and differentiation have been successfully employed to generate organoids that represent different respiratory compartments, such as proximal and distal airways and alveoli (Tan et al., 2017; Zhang et al., 2025a; Nikolic et al., 2017; Chan et al., 2022). The complexity of organoids represents an excellent tool for studying the orchestrated interactions of multiple cell types and tissue-level responses to a fibrogenic stimulus (Strikoudis et al., 2019; Surolia et al., 2017; Joo et al., 2024). To accurately model lung diseases, organoids can be co-cultured with patient-derived immune cells such as AMs that are integral to initiating the inflammatory cascades and immune cell infiltration culminating in the development of silica-induced fibrosis (Vazquez-Armendariz et al., 2020; Harter et al., 2025). It is also increasingly acknowledged that the respiratory immune system plays a fundamental role in maintaining epithelial barrier integrity and lung homeostasis (Weinstock et al., 2021). Incorporating immune cells or humoral immune components into lung organoids should be considered for better mimicking of silica-induced disease (Jose et al., 2020; Harter et al., 2025). To date, only one study has explored silica-induced pulmonary fibrosis using an organoid model (Zhou et al., 2023). This study demonstrated that silica particles potently inhibit lung organoid development by inducing pyroptosis, suppressing cell proliferation and epithelial cell differentiation, and reducing stem/progenitor cell markers such as SOX2 and SOX9 (Zhou et al., 2023). However, in this study immature organoids with apical (ciliated) surface facing inwards were used as opposed to outward orientation. Organoids typically maintain an internally orientated ciliated apical surface, and this orientation may pose challenges for silicosis research. Inhaled silica should interact with the apical surface, necessitating methods to reverse the epithelial polarity in order to face the apical surface outward. The authors of the aforementioned study speculated that apical-basal polarity may influence cell vulnerability and damage, as silica stimulation of apical differentiated cells or basal stem/progenitor cells yielded different responses (Joo et al., 2024; Stroulios et al., 2022). Although use of organoids in silicosis research is lacking, this modelling system has been used effectively to study pulmonary fibrosis when exposed to agents such as TGF-β1 and bleomycin to reflect pathological features such as inflammatory signaling, immune infiltration, and collagen deposition (Tan et al., 2017). These organoids have also been used to evaluate therapeutic potential of novel drugs and the successful induction of fibrosis in organoids highlights their potential in advancing research on silica-related diseases (Surolia et al., 2017; Song et al., 2018; Joo et al., 2024; Sachs et al., 2019). Lung organoids can also be cultured for extended periods, and these constructs can be cultivated for up to a year with no changes in their karyotype whilst maintaining donor characteristics (Sachs et al., 2019; Nikolic et al., 2017). This enables the development of long-term exposure models to study the development and progression of silicosis encompassing the early-inflammatory phase through to the pathogenic remodeling stage. Organoid technology also holds significant potential in personalized medicine. For instance, airway organoids generated from patient-derived epithelial cells from bronchial or nasal brushings, with lung fibroblasts, and endothelial cells, such as those reported by Tan et al., may be used to create patient-specific *in vitro* models (Tan et al., 2017). iPSC derived from skin cells could also be utilised to retain the genetic and epigenetic profiles of patients (Zhang et al., 2024; Heinzelmann et al., 2024). Given the proposed association between genetics and silicosis severity or risk of disease progression, use of patient-specific organoids expose to silica can provide insights into the role of genetics in silicosis development, as well as providing a personalised platform for therapeutic selection (Wang et al., 2018).

Despite the promising potential of airway organoids in furthering our understanding of silicosis pathogenesis, their clinical translatability remains hindered by several key challenges. Traditional lung organoid protocols rely on matrigel and serum-based media, contributing to variability and limiting reproducibility (Zhang et al., 2025a). Organoid maintenance is also resource intensive, technically challenging, and difficult to scale for high-throughput purposes (Andrews and Kriegstein, 2022). While AMs are widely accepted as major players in the acute response to silica, the role of other immune system components is not well known (Gilberti et al., 2008; Li et al., 2025). For instance, the role of conventional Siglec-F^-^ and fibrotic Siglec-F^+^ neutrophil infiltration in silica-induced fibrosis was recently described by Lam and colleagues (Lam et al., 2025). Various immune system components, encompassing both innate and adaptive immune response, should be incorporated to reflect the full physiological complexity of silicosis disease onset and progression.

## 4 Advanced technologies for *in vitro* modelling of silicosis

### 4.1 3D bioprinting

Rapidly advancing in recent years, bioprinting enables automated fabrication of complex 3D-structured tissue models. Representing a powerful technique for scaffold fabrication, it allows the creation of constructs reflective of biological tissue with complex external and internal architectures, depositing material in highly specified locations, and capable of incorporating a number of materials, bioactive molecules, and cell types (Kim et al., 2023; Grigoryan et al., 2019; Kang et al., 2022; Bittner et al., 2018). Precise and controlled computer-assisted deposition of cell-laden bioinks allows for a higher level of spatial complexity with automated high-resolution technologies. The development of 3D bioprinters has accelerated scalability, reproducibility and precision architecture for spatial arrangement using ECM, mitigating many of the challenges associated with classic hydrogel models. Critical to advancing 3D bioprinting for silicosis models will be the choice of bioink, and cell type selection. Bioinks are the building blocks for engineered airways and lung tissues and need to recreate specific functional properties and biological activities in bioprinting in order to reflect the mechanical strength and viscoelastic behaviour of the lungs. Furthermore, different bioink formulations can be used to represent various stages of disease. Bioink formulation and choice for tissue engineering and *in vitro* modelling of the airways and lungs was recently discussed in depth by Zhang et al. (Zhang et al., 2025b). Furthermore, advancements in bioprinting through incorporation of vasculature are being explored (Grigoryan et al., 2019).

3D printed human airway replicas have been used to study the lung deposition of aerosols from different sources such as cigarette smoke and PM, providing more physiologically relevant models for studying respiratory hazards, mimicking the microstructures of the airways. Chen et al. developed a ventilated artificial lung system using 3D-printed airways with active breathing using a ventilator with inspiratory and expiratory limbs and elastic lung balloons to model inhalational exposure of electronic cigarettes (Chen et al., 2024). Such models can be used to inform on the kinetics of silica particles deposition in the lungs and the influence of particle size and co-exposures such as heavy metals and LPS (Ramkissoon et al., 2025; Hetland et al., 2001). The effect of temperature and humidity in the environment on silica deposition can also be examined using these models, which has not yet been reported and can be highly variable across different occupations at risk of silica exposure (Jay and Brotherhood, 2016).

### 4.2 On-A-chip technology and microfluidics

**FIGURE 1:**
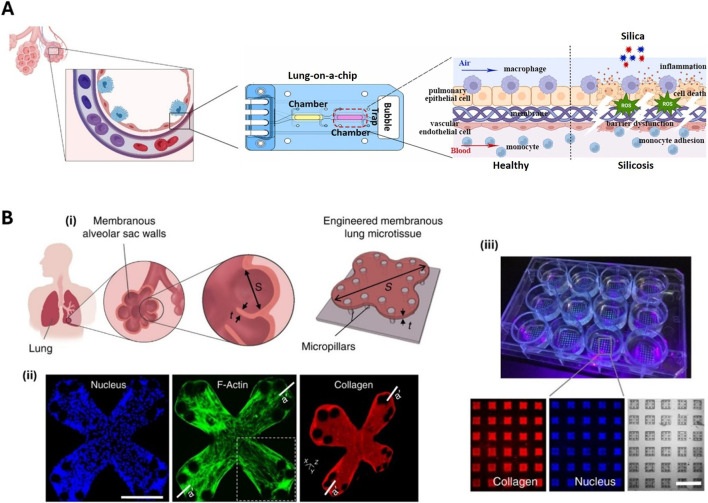
Advanced technologies for modelling fibrotic respiratory disease enabling assessment of pathobiology and drug screening. **(A)** Design and model of lung-on-a-chip with cross-sectional view of two distinct channels separated by a thin, porous membrane with an air interface for modelling exposure to airborne pollutants such as respirable silica particles. Reproduced and adapted with permission of Elsevier, from Yang et al., 2023, permission conveyed through Copyright Clearance Center, Inc. **(B)** Membranous human lung microtissues arrays allow modelling of key biomechanical events during lung fibrogenesis including progressive stiffening and contraction of alveolar tissue, decline in alveolar tissue compliance and traction force-induced bronchial dilation; (i) A lung alveolar sac wall featuring a large surface area and small tissue thickness; (ii) Fluorescent confocal images of an experimentally-created four-leaflet lung microtissue stained for nucleus, and F-actin stress fibers, and collagen type I; (iii) Immunofluorescence imaging of a microtissue array allowing multi-parameter, phenotypic analysis of anti-fibrotic drug efficacy. Reproduced and adapted with permission under a CC BY 4.0 license from Asmani et al., 2018 (https://creativecommons.org/licenses/by/4.0/).

Lung/Alveolus-on-a-chip devices represent a significant advancement in biotechnology that simulate the dynamic components of the respiratory system, blood circulation, and immune system. These devices combine cells with microfluidic technology to replicate physiological and microenvironmental cues such as air-fluid flow, mechanical stretching, hydrostatic pressures, and shear stress (Baptista et al., 2022; Nawroth et al., 2023; Sengupta et al., 2025). Typically containing two parallel channels separated by a porous membrane, the upper chamber is lined with lung epithelial cells and the lower channel is lined with endothelial cells (Figure 1). Cyclic mechanical strain using a vacuum around the channels mimics lung expansion, and air flows through the epithelial channel, while nutrient rich ‘blood’ flows through the endothelial side. By mimicking the thin alveolar barrier and respiratory movements, the system offers a precise *in vitro* model of lung mechanics. Lung chip models can be developed to incorporate a variety of cell types with specific spatial location depending on the research question. Previous studies have used lung chip systems to model various lung pathophysiologies in response to environmental pollutants, monitor fibrosis in response to TGFβ, and assess the efficacy of anti-fibrotic agents pirfenidone and nintedanib (Hayward et al., 2021; Li et al., 2024b; Yang et al., 2023).

A biomimetic lung chip device developed by Goksel et al. has been used to study silica-induced epithelial barrier disruption, whereby Calu-3 human airway epithelial cells were seeded onto a membrane at ALI, and the device was operated under static and dynamic conditions (Nawroth et al., 2023; Sengupta et al., 2025). Dynamic conditions allowed mimicking of particle flow of inhaled silica in the respiratory tract through the epithelium and into the bloodstream. Silica-induced disruption of epithelial barrier integrity was significantly higher under dynamic conditions compared to static conditions, highlighting the importance of physiologically relevant culture systems when evaluating the toxicity profile of silica (Goksel et al., 2024). The importance of dynamic flow conditions on silica-induced toxicity was also reported in other studies, where silica-induced ROS production by epithelial cells required cyclic strain and was not observed in static cultures (Huh et al., 2010). These studies have established the use of lung chip systems for measuring barrier integrity as a measure of silica toxicity. Future studies incorporating various tissue resident cells and immune system components will generate more relevant silicosis-specific models, and allow investigation into additional parameters, such as tissue stiffening and diffusion of silica into the systemic circulation.

Integration of multiple immune cell types into lung-on-a-chip devices has been developed for toxicological application of environmental pollutants such as nanoplastics and cigarette smoke in the context of COPD (Li et al., 2024b; Yang et al., 2023). This allows recapitulation of the structural features and functions of the airway-blood barrier, in addition to the immune responses in the lung, including the activation of neutrophils in response to inflammatory signals or bacterial infection, simulating the process of immune cell infiltration into the alveolar space (Li et al., 2024b; Yang et al., 2023). Although not yet widely investigated for studying the pathobiology of silica-induced disease, lung-on-a-chip devices hold promise for surpassing current 3D culture methods in accurately replicating the complex mechanisms underlying silicosis.

The main disadvantage of lung-on-a-chip technologies is that these are standalone devices, making scale-up for high-throughput usage challenging, and initial manufacturing is labour-intensive and expensive (Francis et al., 2022). However, advancements such as *in situ* force sensors have been developed to detect the changes in the mechanical properties in response to pollutants in order to measure tissue stiffening as a measure of fibrosis development (Li et al., 2024b; Yang et al., 2023; Zhao et al., 2024; Sengupta et al., 2025). Advances in automated on-a-chip manufacturing with integrated sensors for real-time measurement of extracellular metabolites and tissue stiffening may streamline the reproducibility, accuracy, and scalability of these platforms for research into silicosis pathophysiology and drug screening. Furthermore, ECM components are lacking in microfluidic constructs which eliminates the tissue remodelling aspect of fibrosis, and integration with other techniques to generate next-generation models may be required to capture the complexity of silicosis disease.

### 4.3 Integrated 3D platforms

Multiplexing enables further spatiotemporal and physiological complexity, by combining the attributes of various *in vitro* modelling platforms. Fabrication of complex models incorporating facets of the air-blood barrier, cell-laden ECM with tissue-specific spatial arrangement, and dynamic culture mimicking perfused conditions that allows immune cell integration will yield preclinical models that offer superior clinical relevance (Liu et al., 2025; Zhao et al., 2024). Research teams such as Kim et al. have presented novel approaches to developing alveolus-on-a-chip system by integrating high-resolution bioprinting with microfluidic platforms, in which bioprinted lung tissues are cultured on a microfluidic device that provides perfusion at the ALI, closely mimicking the natural lung environment (Kim et al., 2023; Kang et al., 2022; Kang et al., 2021). Advanced systems such as these allows for enhanced control over flow distribution and enables long-term culture under physiologically relevant conditions, critical for evaluating silica-induced fibrosis. In addition, multiorgan-on-a-chip systems could be leveraged to study the systemic response to respirable silica, which has not been well researched despite the established association between silicosis and development of autoimmune diseases such as scleroderma and rheumatoid arthritis (Hayward et al., 2021). The utility of the models is interdisciplinary, and establishing such models will be of significant relevance in areas of environmental toxicology, occupational medicine, and hazard assessment of respirable materials.

## 5 Looking forward: translational applications of silicosis 3D models

### 5.1 Accelerating early disease detection and identifying targeted treatment options

Early detection of silicosis and other silica-related diseases is critical to enable timely intervention and management, thereby improving patient outcomes and reduced the burden of disease. However, insensitive methods of detection and latency between exposure to symptomatic presentation makes identification of early disease onset challenging. A growing body of research focuses on validating biomarkers by assessing their ability to indicate exposure, disease, or susceptibility (Ignatiadis et al., 2021). While research into silicosis biomarkers using blood, urine, saliva, and exhaled breath condensate holds immense promise in screening at-risk workers, much of these initial studies have incorporated patients with established disease due to challenges in accessing samples of those verified as early disease stages (Yao et al., 2024; Ribeiro et al., 2024; Altindag et al., 2003; Chu et al., 2020; Chu et al., 2014). Therefore, validation of molecular markers relevant specifically to the early disease stage may be challenging. Developing improved pre-clinical models of silicosis using the platforms discussed in the previous sections that represent the hallmarks of respirable silica-induced lung disease offer a valuable platform to deepen our understanding of disease-specific pathophysiology, allowing in-depth identification of the molecular drivers of disease onset and progression. Supplementing clinical sample biomarker studies with pre-clinical modelling methods could strengthen predictive validity of identified biomarkers, monitoring expression dynamics from initial silica exposure through to fibrotic remodelling stages.

There is currently no curative treatment for silicosis other than lung transplantation, and management options focus principally on relieving symptoms, preventing disease progression and improving general condition (Li et al., 2023; Handra et al., 2023). Anti-fibrotic therapeutics may be prescribed to patients with silicosis, but there is no established evidence base for this use, and they do not offer restoration of lung function or reversal of lung fibrosis, and gastrointestinal adverse effects may limit compliance (Li et al., 2023; Cavalin et al., 2019; Handra et al., 2023). Lung transplantation may offer a life-saving option for end-stage disease, however this is not a feasible option for most silicosis patients, not least due to the high procedure costs, limited availability of donor tissue, and requirement for long-term immunosuppression (McEwen and Brodie, 2021; Niroomand and Lindstedt, 2025; Redondo et al., 2014). This unmet need for improved pharmacological interventions for patients with silicosis and silica-related disorders ensures poor clinical outcomes including significant symptomatic burden and poor quality of life and impaired wellbeing of patients and their families. Poor clinical success of proposed therapies for pulmonary fibrosis is in part attributed to ineffective pre-clinical testing models, and limited relevancy to human physiology when evaluating therapeutic potential (Dowden and Munro, 2019; Sun et al., 2022). Furthermore, our limited understanding on the drivers specific to silica-induced disease limits development of novel therapies and identifying candidates for drug repurposing.

Developing advanced pre-clinical models again offers a platform to identify and validate drivers of silica-induced disease. Advances in our understanding of silica-induced disease spanning acute injury following initial exposure to progressive fibrotic disease and development of silicotic nodules may enable identification of therapeutics that best suit patients at different stages of disease. Moreover, efficacy assessment of new or repurposed drugs for treating silicosis will require clinically relevant models of disease that recapitulate the feature of disease that is being targeted (Kola and Landis, 2004). For instance, ECM-targeting therapeutics should be evaluated in models that accurately reflect the ECM of silicosis patient lung tissue.

### 5.2 Representing different occupations and silica exposure profiles

There is a significant gap in research examining the biological response to silica using exposure profiles that reflect a wide range of occupational cohorts, specifically with respect to the type of silica, surface chemistry, particle size, concentration, and frequency of exposure. The majority of studies to date use a single, high concentration of silica to examine the response in target cells that does not go beyond the acute phase, and do not incorporate low dose, repeated exposures that are more representative of real-world experiences. Beyond artificial stone manufacturing, emerging at-risk occupational cohorts include tunnelling, quarrying, road construction, asphalt milling, hydraulic fracturing, monumental masonry and stone restoration, ceramics manufacturing, and dentistry, among others. It is important to note that exposure profiles between these settings will vary significantly in nature, including silica particle size and chemistry, frequency of exposure, and co-exposures (including volatile organic compounds, heavy metals, LPS) to name a few (Liu and Sayes, 2022; Chen et al., 2015; Tan et al., 2020; Hua et al., 2023). Using physiological and advanced models that can be maintained for several weeks to months, studies should investigate the long-term effects of silica exposure, incorporating the various parameters that may be unique to particular occupational settings. Evaluation of the harmonious effects of these varying parameters on human health and disease should be evaluated to aid in establishing exposure limits and identifying improved PPE. However, without appropriate sample characterisation and reporting, translation of these research findings into practicable exposure regulation and safety assessments will be challenging. It is suggested by the authors that pre-clinical investigations into different silicates and silica-containing materials incorporate multi-parameter reporting (such as particle chemistry, size distribution, deposited particle dose, sample preparation and storage, *etc.*), to ensure translational relevancy and reproducibility of research findings (Miller et al., 2023).

There is also limited research on the ‘by-stander effect’, where people indirectly exposed to silica dust are at risk of developing silica-related lung diseases without directly handling silica-containing materials, such as family members, health and safety officers, and civil engineers (Bhagia, 2012). In the context of asbestos, the risk of bystander exposure is acknowledged; however, asbestos and silica dusts are significantly different. Workers can carry asbestos fibres home by way of their clothes, vehicles, skin and hair, and meta-analyses have shown family members of exposed workers had high concentrations of asbestos fibres in their lungs and lung tissue abnormalities associated with an increased risk of mesothelioma (Goswami et al., 2013). Similar studies to investigate the bystander effect in silica-induced diseases remain relatively unexplored, underscoring an important gap in occupational and environmental health research. Advanced *in vitro* models examining the effects of silica particle sizes which may be carried home by workers using silica from various source materials at low chronic exposure levels may also provide insight into non-occupational exposure risks.

### 5.3 Toxicity testing of new materials

Aside from the aforementioned gaps in silicosis research, there is a need for robust toxicity and risk assessment models as new ‘silica-free’ and ‘low-silica’ materials arrive on the market for artificial stone substitutes. Given the variable latency to symptomatic disease onset, screening of materials in relevant models should allow for improved toxicology assessments and predictive validity for new material safety profiles. Ruijter et al. recently detailed the use of ALI for hazard screening of silica particles, but noted the limitations of exposing the model to a single, acute (48 h) exposure (Ruijter et al., 2025). In their study, DQ12 demonstrated limited *in vitro* response, while *in vivo* data rank the material as a high hazard due to chronic and persistent inflammatory response, highlighting a functional discord between models. The authors suggested that the health effects observed are likely caused by chronic and persistent exposure, and various endpoint assessments may be critical to produce a robust pre-regulatory hazard screening strategy (Ruijter et al., 2025). It is essential that toxicity assessments of silica-containing materials and studies into subsequent pathobiology account for accumulated toxicity over time, and not solely based on a single, short-term exposure. As discussed in the previous sections, the toxic activity of silica dusts is extremely variable depending on their source and preparation methods. Aside from particle characterisation and reporting requirements, a silica standard reference material should be incorporated to provide a consistent benchmark for toxicity testing of new silica-containing materials. Implementation of reference materials across will enable consistency, rigor, and reproducibility across studies, supporting meaningful and translational impact (Miller et al., 2023).

Aerosolization systems represent a more accurate simulation of occupational exposure and allow dose-controlled repeated exposure over longer cultivation times (Barosova et al., 2020; Chen et al., 2024). Although it may not be feasible for all research centres to obtain an aerosolization system and not all pre-clinical models will allow for aerosolised deposition, improved understanding of the differences in toxicity profile between silica in suspension compared to freshly fractured dust will aid in assigning hazard risks to materials as well as our understanding of silica-induced disease (Rossner et al., 2019; Wallace et al., 2025).

### 5.4 Evaluating the risk of respirable amorphous silica

Although silicosis is predominantly associated with exposure to respirable crystalline silica, the potential hazard of amorphous silica, particularly in chronic exposure settings, has been documented in the medical literature. While generally regarded as less toxic, there is substantial evidence that various types of amorphous silica poses risks to human health and also alter the absorption and metabolism of other environmental pollutants (Dal-Cheri et al., 2025; Guo et al., 2016; Maser et al., 2015; Perkins et al., 2012; Shi et al., 2025; Dong et al., 2020; Lee et al., 2020; Guo et al., 2017). Studies have shown chronic exposure to amorphous silica nanoparticles at low dose exhibits carcinogenic effects in human lung epithelial cells and induce DNA damage in various cell lines (Guo et al., 2017; Lee et al., 2020). Despite such studies, amorphous silica is widely utilised in various industries and in the manufacture of rubber, paints, cosmetics, biomedicine, and food additives (Tomozawa, 2001). These reports underscore the health risks associated with non-crystalline forms of silica, highlighting the urgent need to investigate the exposure hazards of respirable amorphous silica, an area that remains insufficiently addressed (Merget et al., 2002). Comprehensive evaluation of the biological effects of chronic exposure to respirable amorphous silica particles in relevant *in vitro* models is required.

Recent work by Pavan et al. demonstrated that the toxicity of silica particles is not simply a result of the crystalline structure and instead is mediated *via* the presence of ‘nearly free silanols’ (Turci et al., 2016; Pavan et al., 2020). While nearly free silanols may be inherently present as a feature of silica particles, processes such as grinding and crushing increase the number of nearly free silanols, challenging the assumption that amorphous silica is inherently low-toxicity. Where amorphous silica possesses reactive or nearly free silanol groups due to processing or surface modification, it may also exhibit significant toxicity. Therefore, hazard assessment should consider surface properties, not just crystallinity, further emphasising the need for improved sample characterisation and reporting in future studies.

A critical limitation in current pre-clinical models is lack of transparency regarding the specific type, source, and particle size of silica used. Our literature review revealed that many pre-clinical studies investigating pathophysiology of silicosis employed amorphous silica rather than crystalline forms yet failed to report this distinction. Such misinformation hinders progress in understanding the molecular drivers of disease, because different forms of silica and exposure profiles may activate distinct signalling pathways and pathogenic mechanisms. This in turn may also result in differing pharmacological interventions based on the molecular drivers of disease. Furthermore, artificial stone substitutes designed to reduced crystalline silica content for regulatory compliance may increase the proportion of amorphous silica and other substances. Recent studies evaluating the hazards of such materials suggest cause for concern, recommending further investigation (Mandler et al., 2019; Mandler et al., 2020; Ramkissoon et al., 2025). Rigorous assessment of these materials and non-crystalline forms of silica in robust pre-clinical models will be essential to advance our understanding on their hazards and inform effective risk mitigation strategies. In lieu of reliable epidemiological data to support the long-term safety of respirable amorphous silica, a pro-active approach must be taken to ensure workers are not placed at risk and vulnerable to disease due to regulatory ambiguity and insufficient toxicological reporting.

## 6 Conclusion

The global increase in prevalence of silica-related disease presents significant challenges, particularly due to the limited therapeutic options available and the variable latency period from exposure to symptomatic presentation and diagnosis. This is further complicated by underdiagnosis when an e exposure history is lacking, adding to the underestimation of cases of silica-related diseases worldwide. Advanced 3D modelling simulates the physiological environment of human lungs, allowing researchers to explore different facets of lung biology, disease mechanisms, and drug responses. These platforms represent powerful tools to accelerate silicosis research by providing insights into the underlying disease pathobiology, including critical genes and pathways regulating silica-induced lung dysfunction, using physiologically relevant endpoints. Additionally, these 3D models will offer relevant platforms to assess the toxicity of silica particles and silica-containing materials to improve the safety of high-risk workers. While 3D models continue to emerge as a promising alternative to animal testing, it is important to understand the advantages and limitations of each platform. Integrating the features of ALI systems, ECM scaffolds, and microfluidic devices will enable superior representation of human lung biology. However, vigilance will be required when extrapolating findings obtained from *in vitro* models of silicosis, and reasonable representation of human pathology must be confirmed. A critical approach to model development will be essential to ensure findings are of physiological relevance and carry clinical translatability. Ultimately, selection of the most appropriate 3D model should be carefully considered in the context of silica-induced disease and guided by the specific research objective. Finally, greater rigor in the characterisation and reporting of silica properties will be critical for advancing toxicity assessments, enabling meaningful cross-study comparisons, and ultimately improving the prevention of occupational exposures.
